# Short-term and long-term outcomes of on-pump beating-heart coronary artery bypass surgery in dialysis and non-dialysis patients: a retrospective study in a single center

**DOI:** 10.1186/s13019-024-02662-6

**Published:** 2024-04-30

**Authors:** Chia-Hsun Lin, Yen‐Yang Chen, Yu‐Tong Yen, Patrick Hung‐Ju Kao, Chai-Hock Chua, Hung-Hsing Chao, Ming-Jen Lu

**Affiliations:** 1 Division of Cardiovascular Surgery, Department of Surgery, Shin Kong Wu Ho-Su Memorial Hospital, No. 95 Wenchang Road, Shihlin District, Taipei City, 111045 Taiwan; 2 School of Medicine, Fu-Jen Catholic University, No. 510, Zhongzheng Rd., Xinzhuang Dist., New Taipei City, 242062 Taiwan

**Keywords:** On-pump beating-heart, Coronary artery bypass grafting (CABG), Dialysis, Myocardial infarction, Mortality

## Abstract

**Background:**

This study aimed to evaluate the short-term and long-term outcomes of dialysis and non-dialysis patients after On-pump beating-heart coronary artery bypass grafting (OPBH-CABG).

**Methods:**

We retrospectively reviewed medical records of 659 patients underwent OPBH-CABG at our hospital from 2009 to 2019, including 549 non-dialysis patients and 110 dialysis patients. Outcomes were in-hospital mortality, length of stay, surgical complications, post-CABG reintervention, and late mortality. The median follow-up was 3.88 years in non-dialysis patients and 2.24 years in dialysis patients. Propensity matching analysis was performed.

**Results:**

After 1:1 matching, dialysis patients had significantly longer length of stay (14 (11–18) vs. 12 (10–15), *p* = 0.016), higher rates of myocardial infarction (16.85% vs. 6.74%, *p* = 0.037) and late mortality (25.93% vs. 9.4%, *p* = 0.005) after CABG compared to non-dialysis patients. No significant differences were observed in in-hospital mortality, complications, or post-CABG reintervention rate between dialysis and non-dialysis groups.

**Conclusions:**

OPBH-CABG could achieve comparable surgical mortality, surgical complication rates, and long-term revascularization in dialysis patients as those in non-dialysis patients. The results show that OPBH-CABG is a safe and effective surgical option for dialysis patients.

## Background

Patients on dialysis have high risks for coronary artery diseases and displayed poor prognosis after coronary artery bypass grafting (CABG) compared to non-dialysis patients, including higher surgical mortality, postoperative complications, readmission rate, revascularization, and lower survival [1–5]. On-pump heart-beating CABG is a hybrid surgical approach maintaining stable hemodynamics with the support of cardiopulmonary bypass, but without cardioplegic arrest and aortic cross-clamping [6]. This may lead to lower myocardial injury, therefore is an option for patients with high-risk profiles. However, its outcomes in different high-risk groups remain further investigation. The purpose of this study was to evaluate short-term and long-term outcomes in dialysis and non-dialysis patients after on-pump beating-heart CABG.

## Methods

### Patients

This retrospective study enrolled patients underwent coronary artery on-pump beating-heart bypass surgery in our hospital between 2009 and 2019. Inclusion criteria were: 1) with coronary artery disease; 2) indicated for CABG surgery. No exclusion criteria. All necessary information was reviewed from medical record, including demography, clinical characteristics, and postoperative complications. The study was approved by the institutional review board, and the requirement of informed consent was waived.

### Operative procedure of on-pump beating heart CABG and renal management

Our perioperative management of renal replacement therapy were as follows: 1) low-potassium hemodialysis (HD) for consecutive 3 days before operation; 2) only hemofiltration during bypass; 3) start of regular intermittent HD on the first postoperative day, followed by regular intermittent HD three times a week.

CABG was performed with a median sternotomy. A partial cardiopulmonary bypass was used. The left anterior descending (LAD) artery was anastomosed with left or right ITA graft. The non-LAD coronary arteries were grafted with a sequential saphenous vein graft (SVG). The distal end of SVG was anastomosed to ascending aorta using partially side-clamping of aorta. Intra-aortic blood pump (IABP) was used liberally pre- or post-operatively in patients with hemodynamic instability, unstable cardiac rhythms, or poor LV function.

### Outcomes

Short-term outcomes were in-hospital mortality, length of stay, surgical complications. Long-term outcomes were adverse cardiac events, percutaneous coronary intervention (PCI) reintervention, and late mortality during follow-up. Adverse cardiac events included recurrent angina and myocardial infarction (MI). Late mortality is defined as death during follow-up.

### Statistical analysis

Continuous data with a normal distribution were analyzed by Student’s t-test and are presented as the mean ± standard deviation (SD); continuous data without a normal distribution are presented as the median (interquartile range (IQR)) and were analyzed by the Wilcoxon rank-sum test. Normal distribution of variables was tested by Shapiro–Wilk. Categorical data were analyzed with the chi-square test or Fisher’s exact test and are presented as n (%), as appropriate. Patients were matched as propensity score (PS) with the ratio of dialysis: non-dialysis = 1:1 according to age, male sex, BMI, smoking status, preoperative EF, CCS angina class, diabetes mellitus, hyperlipidemia, cardiac dysfunction, PAOD, liver insufficiency, cerebral deficits, number of grafts, complete revascularization, grafting strategy. Kaplan–Meier plot was performed and used log-rank test to compare late mortality between non-dialysis and dialysis patients. Late mortality was identified as death after discharge, and the follow-up duration was estimated from surgery date to the last visit or death. A two-sided *P*-value of < 0.05 was regarded as statistically significant. Data management and statistical analyses were conducted with SAS version 9.4 software (SAS Institute, Inc.).

## Results

### Demographic characteristics

A total of 549 non-dialysis patients and 110 dialysis patients were included in this study (Table 1). Compared to dialysis patients, non-dialysis patients had significantly higher body mass index, higher proportions of male, current smoker, normal preoperative ejection fraction (EF), Canadian cardiovascular society (CCS) classes I and II, hyperlipidemia, complete revascularization, bilateral internal thoracic arteries grafting, and longer follow-up period. Non-dialysis patients also had significantly lower European system for cardiac operative risk evaluation (EuroSCORE), lower proportions of diabetes, cardiac dysfunction, peripheral artery occlusive disease (PAOD), liver insufficiency, and cerebral deficits. After PS matching, 89 non-dialysis patients and 89 dialysis patients were enrolled in the analysis. The characteristics between non-dialysis and dialysis groups were balanced, except EuroSCORE and follow-up period. Non-dialysis patients still had lower additive and logistic score and longer follow-up period.

**Table 1:** Baseline characteristics of the study population before and after matching

Characteristic	Before matching			After matching		
	**Non-dialysis patients**	**Dialysis patients**	***P*-value**	**Non-dialysis patients**	**Dialysis patients**	***P*-value**
	**(*N* = 549)**	**(*N* = 110)**		**(*N* = 89)**	**(*N* = 89)**	
Age, years	63.00 (57.00–69.00)	62.00 (55.00–67.00)	0.065	62.94 ± 9.57	62.69 ± 9.08	0.854
Male sex	443 (80.69%)	74 (67.27%)	**0.002**	63 (70.79%)	64 (71.91%)	0.868
BMI, kg/m^2^	25.92 (23.46–28.40)	24.58 (22.03–26.44)	** < .001**	24.28 (21.91–27.34)	24.80 (22.43–26.40)	0.671
EuroSCORE^a^						
Additive	4.00 (2.00–7.00)	8.00 (5.50–10.00)	** < .001**	6.00 (3.00–9.00)	8.00 (5.00–10.00)	**0.005**
Logistic	2.59 (1.32–6.21)	8.91 (4.73–17.60)	** < .001**	4.58 (2.24–11.94)	7.62 (4.10–15.20)	**0.010**
Year of surgery			0.123			0.462
2009–2010	71 (12.93%)	9 (8.18%)		13 (14.61%)	7 (7.87%)	
2011–2012	121 (22.04%)	15 (13.64%)		17 (19.1%)	12 (13.48%)	
2013–2014	98 (17.85%)	24 (21.82%)		19 (21.35%)	22 (24.72%)	
2015–2016	105 (19.13%)	26 (23.64%)		15 (16.85%)	18 (20.22%)	
2017–2019	154 (28.05%)	36 (32.73%)		25 (28.09%)	30 (33.71%)	
Smoking status			** < .001**			> 0.999
Never-smoker	354 (64.48%)	95 (86.36%)		75 (84.27%)	75 (84.27%)	
Ex-smoker	71 (12.93%)	4 (3.64%)		5 (5.62%)	4 (4.49%)	
Current smoker	124 (22.59%)	11 (10.00%)		9 (10.11%)	10 (11.24%)	
Preoperative EF, %			** < .001**			0.921
> 50, normal LV function	385 (70.13%)	52 (47.27%)		48 (53.93%)	47 (52.81%)	
40–50, mild LV dysfunction	77 (14.03%)	26 (23.64%)		18 (20.22%)	19 (21.35%)	
30–40, moderate LV dysfunction	52 (9.47%)	21 (19.09%)		18 (20.22%)	16 (17.98%)	
< 30, severe LV dysfunction	35 (6.38%)	11 (10%)		5 (5.62%)	7 (7.87%)	
Postoperative EF, %			0.080			0.455
> 50, normal LV function	370 (67.4%)	60 (54.55%)		48 (53.93%)	50 (56.18%)	
40–50, mild LV dysfunction	83 (15.12%)	24 (21.82%)		17 (19.1%)	20 (22.47%)	
30–40, moderate LV dysfunction	50 (9.11%)	13 (11.82%)		9 (10.11%)	11 (12.36%)	
< 30, severe LV dysfunction	46 (8.38%)	13 (11.82%)		15 (16.85%)	8 (8.99%)	
CCS Angina Class			**0.001**			0.887
I or II	141 (25.68%)	19 (17.27%)		17 (19.1%)	15 (16.85%)	
III	210 (38.25%)	30 (27.27%)		26 (29.21%)	25 (28.09%)	
IV	198 (36.07%)	61 (55.45%)		46 (51.69%)	49 (55.06%)	
Comorbidities						
Diabetes mellitus	307 (55.92%)	78 (70.91%)	**0.004**	55 (61.8%)	62 (69.66%)	0.269
Hypertension	422 (76.87%)	89 (80.91%)	0.354	68 (76.4%)	73 (82.02%)	0.356
Hyperlipidemia	341 (62.11%)	38 (34.55%)	** < .001**	33 (37.08%)	34 (38.2%)	0.877
COPD	19 (3.46%)	1 (0.91%)	0.225	3 (3.37%)	1 (1.12%)	0.621
Cardiac dysfunction	107 (19.49%)	31 (28.18%)	**0.041**	27 (30.34%)	23 (25.84%)	0.505
Renal dysfunction	17 (3.1%)	4 (3.64%)	0.7659	7 (7.87%)	4 (4.49%)	0.350
PAOD	12 (2.19%)	14 (12.73%)	** < .001**	6 (6.74%)	7 (7.87%)	0.773
Liver insufficiency	23 (4.19%)	10 (9.09%)	**0.031**	8 (8.99%)	6 (6.74%)	0.578
Cerebral deficits	48 (8.74%)	20 (18.18%)	**0.003**	17 (19.1%)	17 (19.1%)	> 0.999
Number of grafts			**0.005**			0.969
1	17 (3.1%)	0 (0%)		-	-	
2	45 (8.2%)	11 (10%)		10 (11.24%)	9 (10.11%)	
3	112 (20.4%)	37 (33.64%)		26 (29.21%)	26 (29.21%)	
≥ 4	375 (68.31%)	62 (56.36%)		53 (59.55%)	54 (60.67%)	
Complete revascularization ^b^	485 (89.48%)	89 (80.91%)	**0.012**	73 (82.02%)	73 (82.02%)	> 0.999
Grafting Strategy			** < .001**			0.541
NO ITA	39 (7.1%)	5 (4.55%)		6 (6.74%)	4 (4.49%)	
SITA	352 (64.12%)	93 (84.55%)		75 (84.27%)	73 (82.02%)	
BITA	158 (28.78%)	12 (10.91%)		8 (8.99%)	12 (13.48%)	
IABP use	119 (21.68%)	25 (22.73%)	0.808	26 (29.21%)	17 (19.1%)	0.115
Years of follow-up	3.88 (1.61–7.00)	2.24 (0.73–4.01)	** < .001**	4.57 (1.15–6.84)	2.14 (0.73–4.13)	**0.001**

Significant values are showing in bold

Continuous data without normal distribution were presented as median (IQR) and categorical data were presented as n (%)

*SD* Standard deviation, *IQR* Interquartile range, *BMI* body mass index, *EuroSCORE* European system for cardiac operative risk evaluation, *CABG* coronary artery bypass graft surgery, *EF* ejection fraction, *LITA* left internal thoracic artery, *LAD* left anterior descending artery, *LV* left ventricular, *CCS* Canadian cardiovascular society, *COPD* chronic obstruction pulmonary disease, *PAOD* peripheral artery occlusive disease, *BITA* blateral internal thoracic arteries, *MI* myocardial infarction, *PCI* percutaneous coronary intervention, *RCA* right coronary artery, *LCX* left circumflex coronary artery, *SVG* saphenous vein graft, *IABP* intra-aortic balloon pump

^a^There were 502 patients in the non-dialysis group and 104 patients in the dialysis group. 53 of patients were missing in all study population; 11 of patients were missing after matching

^b^7 of patients were missing in all study population

**Fig. 1:**
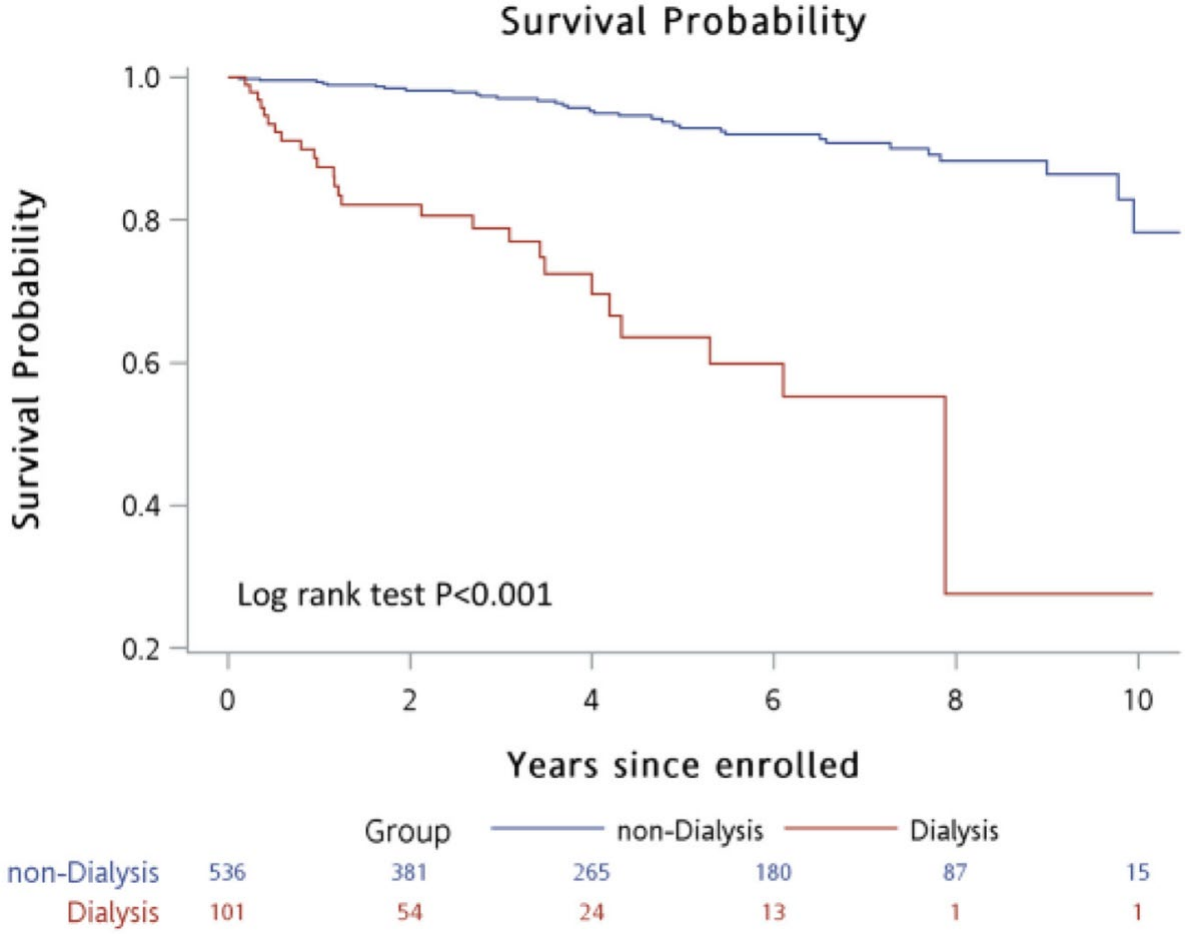
Crude survival rate including all death. A low survival curve was observed in dialysis patients during follow-up compared to non-dialysis patients (Long rank test, *p* < 0.001)

The outcomes after CABG between non-dialysis and dialysis patients after PS matching are presented in Table 2. After CABG, dialysis group had significantly higher longer length of stay (14 (11–18) vs 11 (9–14) days, *p* = 0.016), post-CABG MI frequency (16.85% vs. 6.74%, *p* = 0.037), and mortality rate (25.93% vs 9.41%, *p* = 0.005) than non-dialysis group. No significant differences in in-hospital mortality, surgical complications or post-CABG reintervention were observed between groups. Figure 1 presents the crude survival curves excluded in-hospital death. A significantly lower survival during follow-up was observed in dialysis group compared to the non-dialysis group (*p* < 0.001 with long rank test).

**Table 2:** Postoperative outcomes of the study population after PSM

Variable	Non-dialysis patients	Dialysis patients	*P*-value
	**(*N* = 89)**	**(*N* = 89)**	
**Short-term outcomes**			
In-hospital mortality	4 (4.49%)	8 (8.99%)	0.232
Length of hospital stay for surgery, days	12 (10–15)	14 (11–18)	**0.016**
Surgical complications			
Cardiac	3 (3.37%)	3 (3.37%)	> 0.999
Non-cardiac	26 (29.21%)	24 (26.97%)	0.739
Sepsis	3 (3.37%)	4 (4.49%)	> 0.999
Wound infection	8 (8.99%)	4 (4.49%)	0.232
Respiratory failure	4 (4.49%)	7 (7.87%)	0.350
Hyperkalemia	3 (3.37%)	2 (2.25%)	> 0.999
Gastrointestinal bleeding	1 (1.12%)	2 (2.25%)	> 0.999
Stroke	3 (3.37%)	2 (2.25%)	> 0.999
Reoperation for hemorrhage control	5 (5.62%)	8 (8.99%)	0.387
Requiring ventilation > 24 h	9 (10.11%)	7 (7.87%)	0.600
**Long-term outcomes**			
Postoperative outcome			
Recurrent angina	17 (19.1%)	12 (13.48%)	0.310
Post-CABG MI	6 (6.74%)	15 (16.85%)	**0.037**
Post-CABG reintervention	16 (17.98%)	20 (22.47%)	0.455
PCI for failed LAD graft	6 (6.74%)	4 (4.49%)	0.515
PCI for non-CABG RCA or LCX	7 (7.87%)	10 (11.24%)	0.444
PCI for failed SVG graft	8 (8.99%)	14 (15.73%)	0.172
**Late mortality** ^a^	8 (9.41%)	21 (25.93%)	**0.005**

Significant values are showing in bold

Continuous data without normal distribution are presented as median (IQR) and categorical data are presented as n (%)

^a^Patients died in hospital were not included in the analysis

The outcomes of dialysis patients after CABG are presented in Table 3, including 99 patients with HD and 11 patients with peritoneal dialysis (PD). The in-hospital mortality was 7.07% in HD patients and 18.18% in PD patients (*p* = 0.222). No significant differences were found between the two groups, except the PD group had more cardiac complications than the HD group (18.18% vs 2.02%, *p* = 0.049).

**Table 3:** Postoperative outcomes of the dialysis patients

Characteristic	HD	PD	*P*-value
	**(*N* = 99)**	**(*N* = 11)**	
In-hospital mortality	7 (7.07%)	2 (18.18%)	0.222
Length of stay for surgery, days	14 (11–19)	12 (10–15)	0.081
Complications			
Cardiac	2 (2.02%)	2 (18.18%)	**0.049**
Non-cardiac	28 (28.28%)	3 (27.27%)	> 0.999
Sepsis	5 (5.05%)	0 (0%)	> 0.999
Wound infection	6 (6.06%)	0 (0%)	> 0.999
Respiratory failure	7 (7.07%)	1 (9.09%)	0.582
Hyperkalemia	2 (2.02%)	0 (0%)	> 0.999
Gastrointestinal bleeding	2 (2.02%)	0 (0%)	> 0.999
Stroke	2 (2.02%)	0 (0%)	> 0.999
Reoperation for hemorrhage control	9 (9.09%)	1 (9.09%)	> 0.999
Requiring ventilation > 24 h	9 (9.09%)	1 (9.09%)	> 0.999
Major adverse cardiac events			
Late mortality	24 (24.24%)	2 (18.18%)	> 0.999

Significant values are showing in bold

Continuous data are presented as median were median (IQR) and categorical data are presented as n (%)

## Discussions

The results of the present study, dialysis patients had significantly worse outcomes compared to non-dialysis patients after on-pump beating-heart CABG, including longer length of stay, higher rate of post-CABG MI, and lower survival during follow-up. However, no significant differences in in-hospital mortality, surgical complications, or revascularization rate during follow-up were observed between dialysis and non-dialysis groups. Our results show that on-pump beating-heart CABG is a safe and effective option for dialysis patients.

In the present study, no significant differences in in-hospital mortality, surgical complications, or post-CABG reintervention during follow-up was observed between dialysis and non-dialysis patients. Dialysis is known as an independent risk factor for surgical mortality after CABG [1, 3–5]. Our results showed on-pump beating-heart CABG could achieve comparable short-term surgical outcomes in dialysis patients as those in non-dialysis patients. Meanwhile, Chen et al. reported a higher risk for revascularization after CABG in dialysis patients than non-dialysis patients [3]. It is well-recognized that on-pump CABG ensures comprehensive revascularization [7, 8]. Our result showed that on-pump beating-heart CABG achieves good long-term revascularization also in dialysis group. These results together show that it is a safe and effective option for dialysis patients.

Our results showed that dialysis patients had a significantly longer length of stay, higher rate of MI, and poorer survival after CABG compared to non-dialysis patients. Dialysis is significantly associated with poor outcomes after cardiac surgery [1–5], and the risk for mortality is elevated as dialysis-dependence duration extends [1, 3]. Studies have reported that dialysis patients show significantly higher 30-day mortality, readmission rate, rates of postoperative MI or revascularization, and lower survival after CABG compared to non-dialysis patients [1–5]. CABG is not associated with short-term mortality but improves life expectancy of dialysis patients [5, 9, 10]. For better prognosis, more attention must be paid on dialysis patients’ postoperative conditions after CABG, including monitoring cardiovascular stability, and education of self-care for patients and caregivers.

In the present study, PD patients showed twofold higher in-hospital mortality compared to HD patients without statistically significant difference (2/12 vs. 7/115, *p* = 0.222) probably due to the limited sample size. Studies reported that PD patients were prone to higher in-hospital mortality than HD patients after CABG [11, 12]. Further study is needed to clarify the underlying mechanism.

## Limitation

The study had some limitations. First, it is a retrospective study with those inherent limitations. Second, the study had small sample size and unequal distribution of patients across the dialysis and non-dialysis groups, which may skew the analysis of outcomes. Therefore, PS matching analysis was employed to account for these variables.

## Conclusion

After on-pump beating-heart CABG, dialysis patients have longer length of stay, higher rate of postoperative MI, and poor survival; whereas no significant differences in in-hospital mortality or revascularization rate during follow-up between dialysis and non-dialysis patients. Our findings show that on-pump beating-heart CABG could achieve comparable surgical mortality and good long-term revascularization in dialysis patients as those in non-dialysis patients. It is a safe and effective option for dialysis patients.

## Data Availability

The data analyzed available in the published article.

## Abbreviations

CABG: Coronary artery bypass grafting
HD: Hemodialysis
ITA: Internal thoracic artery
IABP: Intra-aortic blood pump
PCI: Percutaneous coronary intervention
MI: Myocardial infarction
SD: Standard deviation
IQR: Interquartile range
CIs: Confidence intervals
HR: Hazard ratio
PAOD: Peripheral artery occlusive disease
EF: Ejection fraction
CCS: Canadian cardiovascular society
BITA: Bilateral internal thoracic arteries
